# Social contexts as mediator of risk behaviors in Rwandan men who have sex with men (MSM): Implications for HIV and STI transmission

**DOI:** 10.1371/journal.pone.0211099

**Published:** 2019-01-18

**Authors:** Adebola Adedimeji, Jean d’Amour Sinayobye, Brenda Asiimwe-Kateera, Junaid Chaudhry, Lydia Buzinge, Andre Gitembagara, Gad Murenzi, Pacifique Mugenzi, Viraj V. Patel, Philip E. Castle, Leon Mutesa, Joel Palefsky, Kathryn M. Anastos

**Affiliations:** 1 Department of Epidemiology and Population Health, Albert Einstein College of Medicine, Bronx, New York, United States of America; 2 Division of Research and Medical Education, Rwanda Military Hospital, Kanombe, Kigali, Rwanda; 3 AIDS Healthcare Foundation Rwanda, Kigali, Rwanda; 4 Rwanda Nurses and Midwives Union, Kigali, Rwanda; 5 Division of General Internal Medicine, Albert Einstein College of Medicine/Montefiore Health System, Bronx, New York, United States of America; 6 College of Health Sciences, University of Rwanda, Kigali, Rwanda; 7 School of Medicine, University of California, San Francisco, California, United States of America; University of South Florida, UNITED STATES

## Abstract

**Introduction:**

Men who have sex with men (MSM) are disproportionately impacted by HIV/AIDS resulting from risky sexual behaviors. Social and contextual factors are known to mediate risk behaviors, but there is limited information about the prevalence of risky sexual practices of Rwandan MSM and the concomitant socio-contextual determinants making it difficult to assess implications for preventing HIV/STI transmission in this key population.

**Methods:**

Using exploratory qualitative design, we obtained socio-contextual information regarding prevalence of risky sexual behavior and assessed implications for HIV/ STIs transmission and preventive measures taken by MSM to improve sexual health and wellbeing. Thirty MSM were recruited to participate in in-depth interviews using respondent-driven sampling from LGBT associations in Kigali. Data were analyzed using standard qualitative data analysis procedures.

**Results:**

Respondents’ were between 18–40 years old; all completed primary education and are mostly low-socioeconomic status. Risky sexual practices were common, but differed by peculiar individual and contextual factors. Older MSM often reported occasional sexual relations with women to avoid suspicion and social stigma. Younger MSM’s risky sexual practices are mostly transactional and mediated by the need for social acceptance and support. Knowledge of STIs was poor, but prevalence, especially of HPV was high. The options for improving sexual wellbeing are limited and mostly clandestine.

**Conclusion:**

Risky sexual behavior of Rwandan MSM has major implications for HIV/STI transmission. An environment of intense social stigma and social isolation makes it difficult to obtain information or services to improve sexual health. Effective interventions that address individual and contextual determinants of risk and access to health services are urgently needed to limit the consequence of MSM as a bridge for HIV transmission to the general population.

## Introduction

Worldwide, men who have sex with men (MSM) are disproportionately affected by human immunodeficiency virus (HIV), with studies reporting a higher prevalence and incidence of HIV in MSM than that of the general population [1–6]. Studies of MSM in sub-Saharan Africa have observed similar rates of HIV prevalence to be much higher than the average for the general population with some estimates suggesting that African MSM are four times more likely to be infected with HIV than the general population [7, 8].

The Rwanda demographic and health survey data [9] showed that while HIV prevalence remained stable in the general population, there were variations between urban versus rural settings and among sub-population groups. Key populations such as MSM were reported to be at significantly higher risk for HIV acquisition [10, 11] due to widespread risk behaviors- unprotected anal and oral sex, untreated sexually transmitted diseases, pervasive stigma and lack of access to information and services to improve sexual well-being. Similar to other sub-Saharan MSM populations, a considerable proportion of Rwandan MSM also reported sex with women [11], a behavior that can significantly increase the risk of sexually transmitted infections, including HIV, within their sexual networks and the general population [12, 13].

Risky sexual behaviors among MSM are mediated by a host of factors- including individual characteristics [1, 3, 6, 13], type of sexual practices and sexual networks that individuals participate in [14, 15, 16, 17, 18], cultural and contextual factors such as pervasive social stigma against sexual minorities [7, 8, 10], and the lack of specific programs or policies targeting individuals in same-sex sexual relations [7, 10, 19]. These factors not only influence risk behaviors, but also directly limit options for health enhancing behaviors and whether or not individuals have access to information and services that promote sexual health and well-being. Some studies have directly linked patterns of sexual networking and sex partner characteristics to the risk of HIV and STI acquisition in MSM populations [16–18]. Evidence from these studies suggests that in settings where same sex relationships are criminalized and/or stigmatized, it is often difficult to assess and understand the patterns of sexual networking that have implications for HIV transmission and to put in place interventions to minimize susceptibility.

Rwanda is one of 16 countries in sub-Saharan Africa that has not criminalized same-sex relationships. However, pervasive stigma due to religious and socially conservative attitudes, hinder research into the social contexts that facilitate risk behaviors in the transmission of HIV and other STIs. This makes it difficult to develop interventions and policies that promote health outcomes in key populations, a critical group in Rwanda’s strategic goal of preventing new infections and limiting population level impact of HIV/AIDS [19].

Despite extensive documentation highlighting that MSM in sub-Saharan Africa are more susceptible to HIV acquisition due to social contexts that facilitate risk behaviors, very little is known about the social contexts within which risk behaviors are enacted in Rwandan MSM. To our knowledge, there are no existing studies documenting how individual and structural factors mediate risk practices as well as the social factors that impact risk behavior among MSM in Rwanda. The objectives of this study were therefore to highlight and describe the social contexts of sexual risk among MSM in Rwanda and discuss their implications for HIV and STI acquisition as well as health and well-being of this key population.

## Methods

### Study setting

The study was conducted in Kigali the capital city and the largest urban area in Rwanda. We chose Kigali for several reasons: (i) it is the most urbanized setting in Rwanda, (ii) it is the region with the highest HIV prevalence in the country based on results from the Rwanda demographic and health survey of 2015 [20], which shows an HIV prevalence rate of 6% compared with 2% prevalence in rural areas, and (iii) presumably the region most likely to have the largest concentration of MSM population in Rwanda given that urban areas like Kigali may offer opportunities for anonymous sexual interactions between MSM who live in conservative settings compared to rural areas.

### Research design and conceptual framework

We utilized an exploratory qualitative research design to obtain information on Rwandan MSM, sexual behavior, transactional/risky sex and uses of social media/online platforms to recruit sex partners. Using a modified socio-ecological framework, we explored how individual, familial, community and structural/contextual factors shape sexual behaviors, sexual networking and risk taking. This approach facilitated an understanding of how factors that are internal and external to the individual inform decisions to establish and/or sustain sexual relations with multiple sex partners, patterns of sexual networking, uses of social media, health seeking behaviors and implications for the transmission of HIV and other STIs.

### Study population

The study population consisted of self-identified men who have ever had any type of sexual contact with another man in their lifetime and reported to be sexually active within the last 6 months. Sexual contact was defined as any act of a sexual nature, including manual, oral, insertive/receptive anal sex or oral-anal contact (“rimming”). Participants were sexually active males aged at least 18 years, who lived, worked and maintained social ties in Kigali, willing to participate in the study and were able to communicate in either English or Kinyarwanda, the local language widely spoken in Rwanda during the interviews.

### Sampling and recruitment procedures

To recruit study participants, we contacted the leadership of community-based associations of self-identified lesbian, gay, bisexual, and transgender (LGBT) individuals existing in and around metropolitan Kigali. Initial conversations with the leadership about the study and the need to recruit eligible participants revealed two categories of MSM in Kigali- those who are open about their sexual orientation or preferences and belong to existing community-based LGBT associations, and those who are still “closeted” and do not belong to any association because they are yet to reveal or afraid of revealing their sexual orientation/preferences. Based on this information, we recruited participants from the two groups–those who belong to community-based LGBT associations and those who do not belong to any associations.

A three-step modified purposive respondent driven sampling (RDS) strategy [21] was used to identify and recruit a total of 30 males who fit the following eligibility criteria: not less than 18 years, lived in Kigali metropolis in the last 6 months, self-identified MSM, had at least one sexual contact with another man in the 6 months before data collection and willing to participate in the study. In step 1, we identified an initial set of 10 participants who were recruited as “index seeds” (5 among those who belong to associations and 5 among those who do not belong to associations). A two-part study coupon was printed and handed out to index seeds with a request to recruit at least one additional participant into the study from their sexual networks, community organizations, friends or acquaintances who are known MSM. To recruit additional participants, seeds were required to provide contacts with information about the study and contact information of the lead investigator should they wish to obtain additional information about the study. When a seed successfully recruited a participant, they detached the study coupon, handing one part to the new recruit and keeping the other part for reimbursement purposes. Each new recruit who responded to the study invitation provided the part of the coupon that was given to him or her by the seed, which allowed us to identify the index seed that referred them to the study.

Each new recruit was also asked to recruit others in the same way and this procedure yielded a second set of 10 additional participants recruited into the study. We repeated the procedure with the second set of participants to recruit the last set of 10 study participants based on coupons handed out to the second set of 10 study participants. Index seeds and the second set of 10 study participants were each given a primary incentive for recruiting one additional participant and a secondary incentive for participating in the study, a total amount of 12,000 Rwandan Francs (equivalent to $15 USD). The last set of 10 study participants received only the secondary incentive of 8,000 Rwandan Francs ($10 USD) for participating in the study. All the 30 participants that were approached agreed to participate in the interviews.

Prior to data collection, each participant was presented with an informed consent form that explained the purpose of the study, why they were being asked to participate, what kinds of questions they would be asked if they choose to participate, information about incentive and payment as well as possible risks and benefits of participating. When requested, a member of the study team provided more information about the study and informed consent procedures to those seeking further clarification. All recruited participants were required to review, sign and date the informed consent form prior to being interviewed. Informed consent was documented in both English and Kinyarwanda. Ethical approval for the study was obtained from the Albert Einstein College of Medicine Institutional Review Board in New York and the Rwanda National Ethics Committee (RNEC) in Kigali, Rwanda.

### Data collection

Overall, 30 semi-structured individual in-depth interviews were conducted between October and November 2015. Members of the research team fluent in both English and Kinyarwanda conducted the interviews. They were assisted by study staff from the Rwanda Military Hospital and the College of Health Sciences at the University of Rwanda who were experienced qualitative researchers.

A semi-structured individual in-depth interview guide (S1 and S2 Files) was designed and used to obtain information from participants. The semi-structured nature of the guide allowed interviewers to pursue other lines of inquiry that emerged during interviews. The guide focused on specific topics that were determined after a review of literature, information from key informants/leaders of MSM community-based associations as well as individual, familial, community and structural factors that impact on sexual activities and sexual networking of MSM. Specifically, we explored how individual demographic characteristics, social networking, social and community attitudes towards MSM in Rwanda, impact on sexual behavior and access to information and services for sexual well-being among others.

Given the sensitive nature of the topic of same-sex relationships in Rwanda, we took certain precautions in setting up the interviews, ranging from the sex composition of interview facilitators to the location where interviews were held. Each interview was facilitated by a group of 2; consisting of an interviewer and a note taker. As the interviews required participants to disclose sensitive and sometimes uncomfortable information, they were each given an opportunity to select the sex composition of the facilitators, which could include same or mixed sex individuals depending on the preference of the interviewee. Interviews were held at a location that was previously discussed and agreed to by all participants. The interview setting consisted of a single private room in a nondescript building within an environment that allowed participants to feel as comfortable as possible. The average length of the interviews was 60 minutes and participants had a choice of English or Kinyarwanda as the language of interview. Each interview generated 3 outputs- the audio recording, interview notes by the facilitator and interview/observational notes by the note-taker. These documents were reviewed and compared to ensure that they truly reflect all the issues that arose during interviews in addition to filling missing gaps between the written notes and audio recording.

### Data analysis

Data analysis involved an iterative process consisting of 3 steps. The first step involved the initial processing of data, which began with the transcription of audio recordings of each individual interview. All interviews conducted in Kinyarwanda were transcribed verbatim and went through a process where they were first transcribed in Kinyarwanda, then translated into English using the Kinyarwanda transcript and then back translated into Kinyarwanda and verified for consistency and accuracy. Members of the research team at the Rwanda Military Hospital and the University of Rwanda College of Health Sciences did all transcription and translation. Transcripts in both Kinyarwanda and English were independently verified, checked for completeness and scanned to ensure personal identifiers had been deleted. In addition, the authors also reviewed the transcripts several times to become familiar with the data. Following this process, members of the research team, including several authors of this manuscript compiled a list of initial themes, based on issues emerging from the data.

The second step in the data analysis process was the convening of a stakeholders’ meeting involving members of the MSM community who were invited to participate in a data validation exercise. The purpose was to ensure that the research team’s understanding and interpretation of the data is consistent with the reality of the MSM community in Kigali. The three-day workshop, held in December 2016 involved leaders of community-based MSM organizations and other members who did not participate in the data collection. At the workshop, transcripts and the list of initial themes, developed by the 4 of the authors was shared with the participants who were required to peruse the transcript and the list of themes and to suggest if there are additional issues/themes that should be included in the data analysis. This process resulted in a few additional themes that were added to the initial list to generate a comprehensive list of themes that formed the basis for coding of the data.

The final step in data analysis involved finalizing the list of themes and identification of codes, which were then applied to the transcripts. Transcripts verified for completeness, consistency and accuracy were uploaded into QDA Miner [22], an open-source software for analyzing qualitative data. Using this software, inductive thematic text analysis was performed by iteratively reviewing, interpreting and discussing verbatim texts by the research team based on feedback from the data validation workshop. Analysis of the text resulted in an initial set of codes that were independently developed by the lead author. Other members of the research team further independently reviewed this initial set of codes and suggested additional codes, which ensured consensus for a comprehensive and exhaustive list of codes generated. Codes were further matched with the list of themes and any discrepancy was resolved before they were applied to transcripts. We applied the results to an ecological framework to highlight socio-contextual factors influencing sexual practices of Rwandan MSM and whether these are individual, familial, communal or structural in nature. In presenting the results, verbatim quotes from participants were used to illustrate the issue being presented, but we used only the first initial of a pseudonym assigned at the interview to protect their identity.

The consolidated criteria for reporting qualitative research (COREQ) checklist (S3 File) highlight different components of the study methodology.

## Results

### Participants' profile

Table 1 shows the participants demographic and socio-economic characteristics. By design, we recruited half of the participants from LGBT associations and the other half who did not belong to LGBT associations. All participants, regardless of whether they were recruited via LGBT association or not, shared similar characteristics. Respondents were at least 18 years old, with the oldest participant aged 40 years at the time of interviews. All the respondents completed at least primary school level education and about one-third reported completing vocational training and tertiary education. About half of participants reported being unemployed and the other half reported being self-employed or employed by someone else. Regardless of employment status, almost all were of low socioeconomic status considering the reported income of less than 30,000 RWF ($36 US) per month. About half of respondents reported being in romantic relationships with a stable partner and of these, only a few reported that they are currently living with their partners. More than half of participants who were recruited because they do not belong to LGBT associations indicated that they were married or in relationships with women. Half of our sample selection included participants who are members of LGBT associations and half who were non-members. Members of LGBT associations were usually younger, more educated, more open about their sexual orientation, less likely to report being in long term stable relationships, less likely to report having source of income, and less likely to be married to a woman than the non-members of LGBT.

**Table 1:** Participants demographic and socio-economic characteristics.

Characteristics	Number	Percentage
**Age**		
18–25	10	33%
26–35	17	57%
36 and above	3	10%
**Membership in LGBT Association**		
Yes	15	50%
No	15	50%
**Education**		
Secondary completed	20	67%
Vocational Training	7	23%
Tertiary	3	10%
**Disclosure status**		
Openly LGBT	11	37%
Closeted LGBT	19	63%
**Marital Status**		
Single	7	23%
Stable relationship with a man	15	50%
Married to a woman	8	27%
**Employment status**		
Unemployed	15	50%
Self Employed	3	10%
Employed by other	12	40%
**Monthly income**		
Not employed/unknown	15	50%
<30,000RWF	12	40%
>30,000RWF	3	10%

### Determinants of sexual risky practices

Our main objective was to utilize the socio-ecological model to identify and describe the determinants of high-risk sexual behaviors in our study population. Our data revealed several factors at individual, familial/community and larger societal/structural levels that influence risk behaviors.

### Micro-level interpersonal and individual factors

#### Knowledge of HIV/AIDS and other STIs

The majority of participants reported awareness of HIV/AIDS as a sexually transmitted infection linked to unprotected sexual activity. The near universal awareness of HIV/AIDS did not reflect on awareness of other sexually transmitted infection, including gonorrhea, syphilis, chlamydia and anal warts. When probed to mention other STIs known to them, only half of participants reported awareness of common STIs including gonorrhea, syphilis and anogenital warts. Participants who reported awareness of an STI indicated their source of information is based on personal experience or knowledge of someone who was previously reported or suspected to have contracted a specific STI.

The proportion of participants reporting awareness of HIV/AIDS and other STIs mask the low level of knowledge of STIs other than HIV/AIDS. For example, several participants mentioned “*hearing about anal warts*”, an infection commonly associated with human papillomavirus (HPV) infection, but none of the participants “*know that anogenital warts have implications for HPV and/or HIV infection*”.

#### Sexual relationships and risk behaviors

Participants' sexual relationships were of 2 types: those in relationships with a steady partner and those without steady partners, but who are involved with long-term casual partners. Relationship type differs by age, education and socioeconomic status. The combination of demographic characteristics, socio-economic status and relationship status largely impact on the dynamics of risk-taking sexual behavior.

All the participants reported multiple lifetime sexual partnerships and about half reported multiple sexual partnerships in the 6 months prior to data collection. Half of participants who are non-members of LGBT associations reported multiple concurrent sexual relations with both men and women. Younger, more educated participants who are more open about their sexual orientation reported sexual relations with multiple male partners concurrently. Older participants who are less open about their sexual orientation are however more likely to report being in stable relationships with a steady partner, usually a woman to whom they are married so as not to raise suspicions about their sexual preferences for men. They are also more likely to have concurrent sexual relations with both male and female sexual partners.

Unprotected sexual behavior with both men and women vary by individual characteristics and the length of a relationship. For instance, among those who reported ever having had sexual intercourse with male partners, the decision to use a condom or take other preventive measures during sexual encounters is predicated on the length of the relationship or how well known/trusted is the sexual partner. Two of the participants’ statements illustrate the dynamics of risk-taking in sexual encounters:

"*When you newly meet someone and you need to have sex*, *you are more conscious about protecting yourself as you don’t know with whom they have been and whether or not they have any disease you have to prevent yourself from*" (A, 26 years old, Open)

"*Your decision to use any protection will depend on how long you have known the person*. *For instance*, *I started out using condoms with my partner when we first met*, *but as time goes on and we got to know each other better*, *it was no longer necessary to use condoms*. *Although*, *I know that you also have to trust the person and be sure they are not cheating on you to be absolutely confident not to use any form of protection*" (K, 30 years old, Open).

Among those who reported sexual activity with both male and female partners, the decision to use a condom or other preventive behaviors is similar but somewhat different depending on the type of partner involved. For instance, those who reported marriage to women almost never use condoms when they have intercourse with their wives because they want to "*avoid suspicion of any extra marital activity*". However, in sexual encounters with other men, the decision to use any protective measure also depends on the type of partner, the length of time and whether or not it is a transactional sexual encounter. As one participant reported:

"*It is difficult to use condom with your regular partner because that will raise suspicion that you have been unfaithful or the feeling that you don’t trust them*. *But when you have sex with someone else*, *whether a man or a woman*, *you want to protect yourself and be sure you don’t get any disease*. *But if it is someone I trust*, *I will not use a condom because I won't enjoy sex that way*"- (C, 36 years old, Closeted)

The processes in recruiting sexual partners also highlight the social contexts of risky behaviors. Members of the MSM community in Kigali are still very discreet about their sexual orientation, therefore they have limited opportunities to meet or socially interact. Consequently, opportunities for meeting and recruiting sexual partners are limited to a small network of friends introduced by other MSM during "*house parties*" organized to facilitate social interaction. In some cases, social media and online dating platforms are used to recruit sex partners and initiate sexual relations that are transactional in nature, often involving the exchange of money or gifts. Participants who reported these methods for recruiting new sexual partners indicated that the decision to take any protective measure is mediated by the circumstances of the meeting, the place or time and conditions under which sexual intercourse is negotiated. As reported by some participants:

"*Ours is a small community and most people are still afraid to show who they are*. *You are always careful because you don’t want to expose yourself to danger if people know you are gay*. *So you rely on your friends to introduce you to someone they know and trust and in that case the decision to use protection when sex happens is up to the two people*" (B, 27 years old, Closeted)

"*When we organize house parties*, *everyone knows it is an opportunity to meet new people*, *have fun and sex*. *Because a lot of alcohol is consumed at house parties*, *a person's sense of judgment may be impaired and therefore does not consider the risks involved in unprotected sex* …*that is the last thing on your mind*, *you just want to have a good time* …" (B, 24 years old, Open)

"*I know of friends who use social media and online dating platforms to meet other MSM and find sex partners*. *Sometimes*, *this involves exchange of gifts or money and when you are being paid to have sex*, *you cannot control whether or not you protect yourself*. *It is* [mostly] *up to the person who is paying you*." (K 30 years old, Open)

### Meso-level social, family and community factors

#### Impact of social stigma

An environment of intense stigma created situations in which some of the participants reflected on how stigmatizing attitudes affect their relationships with family, friends and the larger community. Our data show that those who perceived that they lack close relationships with family and community members and/or who feel stigmatized by those they feel should accept them regardless of their sexual preferences are more likely to indulge in risky sexual activities. To compensate for the loss of familial relationships and attenuate the effects of perceived stigma, some MSM become involved in relationships with what they described as "*people like me*, *who need no explanation for who*, *what or why I am what I am*". For example, only few of the participants reported that close family members are aware of their sexual orientation. For many others, the fear of rejection and deprivation from close family members warrants keeping their sexuality a secret and refraining from close connection with anyone who can potentially "*raise the uncomfortable question of why don’t you have a woman in your life*?" Those who have previously taken the step to let close family members know their sexual orientation reported reactions ranging from shock to outright rejection and being sent away from the family home. One participant shared his experience:

"*My parents have always been suspicious of me* …*that I behave too much like a girl*. *One day*, *I decided to let them know and it is a decision I have regretted*. *They not only told me I will no longer be able to live with them*, *they stopped communicating with me*. *At the moment*, *I have no close family I can talk to when I am in trouble*" (B, 24 years old, Open)

And another indicated that such situations mean you only have people within the MSM community to rely on when he said:

"*This kind of situation when your family refuse to accept you the way you are means the only family and friends you have are other MSM who have accepted you the way you are*. *Why would you not want to have as many friends as possible even if there are certain risks involved*? *You do whatever it takes to keep and not lose them*" (L, 29 years old, Open)

Tense relationships with other (non-family) community members had a similar effect- the feeling of social isolation- on individual MSM. Due to cultural and conservative beliefs, participants feel many Rwandans do not show empathy towards, appreciation for, or understanding of what they considered "*sinful behavior*" of MSM. Consequently, many MSM are unable to disclose their sexuality or indeed socialize without fear of suspicion or exposure to the risk of prejudice and personal injury. For example, the consensus from participants is that when MSM need to avail of services to improve their health and well-being, perceived and enacted stigma from community members makes it nearly impossible to access services that are often not available. Two participants describe their experiences:

"*You really have to be careful how you behave when you are in public*. *Many of us [MSM] are afraid of what will happen when someone finds out about us* [our sexuality]. *We know the risk associated with being MSM in some places and we sometimes don’t feel safe*" (L, 29 years old, Closeted)

"*There was a time I was sick and needed to see a doctor*. *I was very afraid*, *asking myself do I go with this problem and say I had sex with a man*? *It took a long time to summon courage to see a doctor and that was after a friend recommended a doctor they have used in the past and is well trusted by MSM*" (K, 30 years old, Closeted)

In an environment where MSM are distrustful of family and community members, a close-knit friendship/sexual network has developed within the gay community in which members rely on one another to establish romantic relationships, and obtain critical resources to navigate a very conservative cultural and social environment to ensure they have access to jobs, legal and social services, and general health and well-being. Most participants however believe that strict societal attitudes and conservative perspectives are changing because MSMs are becoming more open, more active and have succeeded in pushing back efforts to legislate against or criminalize sex relationships. More importantly, the effects of global activism and receptive attitudes towards LGBT in western societies that have become known to Rwandans due to media coverage in recent years is slowly changing the social environment in Rwanda.

Regardless, many participants indicated a change is necessary within Rwandan society regarding the rights of MSM and LGBT people. They suggested that such changes need to start with family members accepting their sons and loved regardless of their sexual orientation.

### Macro-level structural factors

Macro-structural factors that increase the susceptibility of MSM to risky sexual behaviors mostly occur at the policy and program level. For example, participants reported that the lack of programs or health services specifically targeting sexual health of MSM also contribute to their vulnerability. They mentioned the lack of state-supported opportunities for social interaction among MSM and complained that Rwanda is unlike other western societies where a gay couple can go out in public and not be ashamed to hold hands and show affection for each other, a situation they attributed to the failure of political leaders to actively show support for the gay community. One participant, who is an activist in one of the LGBT groups, indicated that:

"*Although*, *there are no specific legislation against MSM*, *the silence of political leaders to openly talk about the rights and privileges of LGBT people contribute to the stigma and inaction to care more about our community*. *If our leaders are more open in their opinions and publicly show support*, *the rest of the society will follow*. *As you know*, *Rwanda is a country where political leaders have a lot of influence over the population because of the high regards we have for them*" (P, 30 years old, Open)

Macro level structural factors that impact the lives of Rwandan MSM also manifests at the level of the health system. As one participant put it:

"*There is not a single hospital in Kigali where MSM can go to access services with the confidence that someone there understands the health issues affecting us*. *Even when you have the courage to go to a hospital*, *you are unable to say who you are because of the stigma they will put on you*. *Many people therefore prefer to treat themselves*, *which worsens the situation because whatever you try may not be effective*. *It is not easy for MSM in this country*" (P, 30 years old, Open)

Another respondent describes the nature of clandestine health services that are accessible to most MSM:

"*I know a lot of people who are unable to go to government or private hospital to complain about their health issues because they are afraid and no one wants to deal with them because of their sexuality*. *For most people*, *the option is to self-medicate based on videos and information from the internet or friends*. *Some of us are lucky to know members of our community who are doctors and we meet them for advice and treatment but they too get overwhelmed at times and are not available* …" (B, 24 years old, Open)

The lack of MSM specific sexual health services, stigmatizing and discriminatory attitudes from health workers and non-availability of personnel with relevant training to handle peculiar health needs of MSM were also cited as reasons for not utilizing mainstream health facilities. For example, one participant described the difficulty with accessing health services when he said:

"*There was a time I had this problem and it was getting worse*. *I knew I should go and see a doctor but I was afraid and confused because I don’t know how they will react*. *Can I go and say it is because I had sex with a man*?" (J, 27 years old, Closeted)

Despite the challenges associated with accessing health services, several participants indicated that members of the MSM community usually access health services provided by few doctors who are either MSM themselves or are sympathetic to the plight of MSM.

The lack of policies, programs and political will demonstrating support for MSM also hinders research documenting the situation of Rwandan MSM and collaborative work among civil society entities willing to develop and implement interventions addressing health, social and financial issues facing MSM. One participant described the feelings of many Rwandan MSM when he said:

"*Unlike in other countries with lots of research on MSM and civil society organizations working with them*, *the lack information on the challenges and issues faced by Rwandan MSM leaves us feeling that we are not recognized as valuable members of the society*"(P, 30 years old, Open)

Similar assertions were made with regard to the circumstances of young MSM living in rural parts of the country. Many of the participants agree with the opinion that "*life as a young gay man in the rural areas is extremely challenging because you have no access to any social network*, *services or even the few opportunities existing for urban MSM*, *hence many rural MSM are desperately seeking a way to relocate to the urban areas*".

## Discussion

The foregoing demonstrates that the combination of micro-, meso- and macro-level factors critically impact the social contexts and sexual behavior of Rwandan MSM. Understanding this social context in light of risky sexual behaviors and its implications for acquisition of HIV and other STIs is an important area of research given limited information and interventions about sexual minorities in sub-Saharan Africa in general and Rwanda in particular. Using a socio-ecological approach, we describe how several of micro, meso and macro determinants influence sexual practices and attitudes in Rwandan MSM.

Our study highlights the complexity and diversity of sexual relationships amongst Rwandan MSM and the challenging social, cultural and structural environment within which they occur similar to what has been reported in other studies [11, 23–25]. The patterns of sexual behavior, sexual networking and associated risk practices are mediated by social, cultural and structural factors. At the individual and interpersonal level, knowledge of STIs, the context of sexual and romantic relationships, and sexual risk taking all play a role in the health and well-being of Rwandan MSM.

We found that many of the study participants are generally aware of different STIs, including HIV/AIDS, but they demonstrated poor knowledge regarding the symptoms of specific bacterial and/or viral STIs, including anal warts, which is a physical manifestation of human papillomavirus. Some studies [26, 27] have similarly reported poor knowledge of STIs among MSM, thus interventions to increase knowledge of other STIs apart from HIV/AIDS are urgently needed so that individuals can take appropriate preventive methods when at risk or manifest symptoms. Relatedly, sexual risk taking is common as only few participants take precaution to avoid any infection in sexual relations, especially with long-term partners.

The socio-demographic characteristics of Rwanda MSM suggest there are two categories. On the one hand are those that are young, educated, single, of low socioeconomic status and belonging to an LGBT association, and on the other hand are those who are older, with higher socioeconomic status, married to women and do not belong to any LGBT association. Similar to reports by Maskut and colleagues [28], we found that different factors impact on sexual behavior and risk practices of younger when compared with older MSM. Young MSM are more open about their sexual preferences, more likely to have multiple and concurrent male partners and rely on membership in LGBT associations for sexual networking as well as to obtain information about sexual health and well-being. Older MSM, particularly those who are married to women tend to be more discrete about their sexual preferences for and relations with men, tend to have multiple male and female partners and do not belong to or participate in LGBT associations or activities.

Some studies [15, 17, 24] have reported that similar patterns of sexual networking in MSM populations in highly stigmatized settings have significant implications for sexual health that are diverse, complex and fluid. Consequently, effective interventions to promote sexual health and well-being of MSM and other sexual minorities need to consider the complex milieu within which sexual relations are negotiated and enacted.

Communal and familial factors also influence the social context and risk practices of MSM in Rwanda. Our data suggest that intense societal stigma, social isolation and discrimination play a role in individual's susceptibility to engage in risky behaviors, especially as it tends to diminish a sense of self worth and self efficacy. Although, there are no laws criminalizing same sex relationships, Rwanda is still a socially and religiously conservative society where any form of same sex relationships are frowned at. Studies that have examined the effect of stigma and social isolation on men who have sex with men have reported similar findings [29–32]. Parker and colleagues [29] for example explored the concept of social risk and its implications for risk practices among MSM and concluded that social risk shapes risk taking behavior along four domains- (i) where to find sexual partners, (ii) where to engage sexual relationships, (iii) what kind of relationships to seek and (iv) whether or not to use any protective measure, including seeking information and services to improve sexual health and well-being [33].

The meso-level social, familial and community determinants such as strained or broken relationships with family and community members may predispose MSM to seek social acceptance from people who may be sympathetic to their circumstances and may invariably lead to engagement in sexual activities with multiple concurrent partners. Although, Rwanda has been an exception in a region where several countries have passed legislation criminalizing male-to-male sexual relationships and while same sex relationships are not illegal, intense social stigma against homosexual activity continues to negatively impact the health and well-being of Rwandan MSM.

To attenuate the effect of social isolation, many MSM, especially younger MSM form close-knit social networks in order to escape social isolation. These close-knit social networks, which often manifest in the form of LGBT associations, provide psychosocial, emotional and informational support targeted at improving sexual health and well-being of their members. Membership in LGBT associations and social networks also provide MSM an opportunity to advocate for issues concerning them including demanding for access to basic services and healthcare. Consistent with findings from other studies [34, 35] participation in formal or informal social networks tends to have a positive effect on MSM especially in promoting health-enhancing behaviors.

Structural factors at the larger societal level impacting the lives of Rwandan MSM may be more challenging to address if there are no large-scale changes in social attitudes toward and perceptions of people in same sex relationships. As the data show, the lack of interventions that promote health and well-being of sexual minorities or supportive policies and political action creates a barrier for civil society organizations with resources to develop and implement much needed interventions and for MSM to access and utilize services. The social structure in Rwanda, which relies heavily on strong community and familial networks can be harnessed in generating support from community and opinion leaders for community-based health services that cater to the needs of sexual minorities. Research [36–39] has shown that in contexts where there is cultural acceptance of sexual minorities as well as buy in from important stakeholders, interventions are not only less challenging to implement, but can facilitate access to and use of those services.

The limited availability of MSM focused sexual health services in the health facilities in Kigali and elsewhere in the country is indirectly attributed to lack of political will from politicians and leaders. Consequently, most members of the LGBT community resort to using facilities where the health workers do not understand their concerns or respect their sexual orientation or gender identity, thereby forcing many MSM to resort to informal or clandestine health services, which in some cases are provided by people who are unqualified.

Addressing policy gaps require creating an enabling and sustainable environment to change the negative perceptions of people in same sex relationship and willingness to spend political capital from the leaders and other stakeholders. Although Rwanda has responded strongly to the HIV/AIDS epidemic by committing enormous resources to prevention, control and treatment, these efforts would have to include at risk sexual minorities, especially MSM, to be effective and sustainable in reducing the transmission of HIV and other STIs over the long term. To do this will require the identification and involvement of different stakeholders who are committed to changing the current social landscape by openly addressing stigma and committing resources to improve well-being of MSM. The Rwanda HIV/AIDS National Strategic Plan 2013–2018 [40] specifically mentioned men who have sex with men as a vulnerable at risk population in the effort to reduce the spread of HIV. Interventions targeting this group as well as other vulnerable risk groups would be effective if there is a corresponding change toward more positive societal attitudes and acceptance of MSM.

## Conclusion

The social context of MSM in Rwanda, especially in view of the high prevalence of unprotected concurrent and multiple sexual partnerships has major implications for the transmission of HIV and STIs among Rwandan MSM. An environment of intense social stigma makes it difficult to obtain information or services about sexual health and well-being. Program and policy interventions aimed at community advocacy to minimize stigma, education about risk factors for HIV/HPV/STIs and increased access to sexual health services are urgently needed to limit the spread of HIV and STIs in the general population.

## Supporting information

S1 File Interview Guide_Kinyarwanda.doc. (DOC)

S2 File Interview guide for MSM Rwanda.docx. (DOCX)

S3 File CREQ Checklist for Rwandan MSM PLOS One Revision, 2018.pdf. (PDF)
