# Supplementary material for: Social contexts as mediator of risk behaviors in Rwandan men who have sex with men (MSM): Implications for HIV and STI transmission
Source: PLoS One. 2019 Jan 18;14(1):e0211099. doi: 10.1371/journal.pone.0211099 (PMC6338414; doi:10.1371/journal.pone.0211099)
Supplement: S1 File — (DOC) [file pone.0211099.s001.doc]

**KINYARWANDA**

**IKIGANIRO NGENDERWAHO KU BAGABO BAZITABIRA UBUSHAKASHATSI**

Urakoze kuduha umwanya wawe uyu munsi

Amazina yanjye ni: _______________________________.

Ndi umwe mu bashakashatsi bari mu Rwanda, bari gukora ubushakatsi ku bibazo by’ubuzima abagabo bagirana imibonano mpuzabitsina n’abandi bagabo bahura nabyo mu Rwanda. Dushishikajwe cyane cyane no kumenya ndetse no kumva neza ibintu bitandukanye by’imibereho n’imyitwarire harimo n’ikoreshwa ry’imbuga nkoranyambaga ndetse n’ubumenyi abantu bafite ku bijyanye n’ubuzima harimo na virus yitwa Human papilloma (HPV) ndetse n’uburyo abantu bitabira gahunda z’ubuzima muri rusange. Iyo virus niyo itera kanseri y’inkondo y’umura mu bagore ariko no mu bagabo ikaba itera kanseri y‘ikibuno cg igitsina.

Hari ibibazo twegeranije byadufasha kumva ibyo bibazo ndetse cyane uburyo ibyo bibazo bigira ingaruka ku bantu bagirana imibonano mpuzabitsina n’abo babihuje. Turashaka kumenya ukuntu abahungu/abagabo bagirana imibonano mpuzabitsina n’abo babihuje bafashwa guhangana n’ibibazo by’ubuzima n’iby’imibereho myiza bahura nabyo, ndetse no kumya impamvu zishoboka zatuma umuntu yitabira cyangwa atitabira gahunda zo gusuzumwa virus ya papilloma itera kanseri. Uratwihanganira mu bibazo twateguye hari ibyo ushobora kumva bisa n’aho bibangamye gato. Turakumenyeshako ufite uburenganzira bwo kudasubiza ibibazo wumva bikubangamiye, kandi wisanzure igihe waza kumva ushaka ko mpagarara, nuza kumva udashaka ko dukomeza iri bazwa.

Mbere y’uko dutangira, nagira ngo nkumare impungenge ko ibisubizo byose usubiza bazafatwa ku buryo bukomeye bw’ibanga kandi amakuru azakoreshwa gusa mu bijyanye na buno bushakashatsi. Nagira ngo nkubwire ko iri bazwa riri bukorwe mu buryo bukomeye bwo kubahiriza ibanga n’ubuzima bwawe bwite (vie privée) kandi ntawe uzamenya ibyo twaganiriye. Abantu bonyine bazamenya ibikubiye mu byo turi buganire ni abantu bari muri ubu bushakashatsi gusa kandi nabo bakazabikoresha muri gahunda ya buno bushakashatsi gusa. Ndakoresha akuma gafata amajwi y’ikiganiro tugirana kugira ngo ntaza kugira icyo nibagirwa cy’ingenzi mubyo tuganira cyanga nkaza kujya ngucamo kenshi ndi kwandika. Uramutse wumva udashaka ko tuganira nkoresha icyuma gifata amajwi ufite uburenganzira bwo kubimbwira. Niba kandi wemeranya nanjye ku byo nakubwiye haruguru nagira ngo uze kubinyemerera mu buryo bw’amagamo mbere y’uko nkomeza. Nagira ngo uvuge mu buryo bw’amagambo ari bufatwe amajwi ko wemeye ko dukomeza kino kiganiro.

Urakoze cyane.

**TANGIRA IKIGANIRO. ATSA AKUMA GAFATA AMAJWI HANYUMA WIVUGE MU MAGAMBO MACYE, IKIGAMIJWE MURI IKI KIGANIRO UBUNDI UHABWE UBURENGANZIRA BWO GUKOMEZA**

**IBIJYANYE N’IMIBEREHO**

1. Nagira ngo dutangire utwibwira. Ushobora gukoresha izina ryawe nyakuri, irihimbano cg irindi wakwiyita muri iki kiganiro
   1. Wavutse ryari?
   2. Wavukiyehe?
   3. Utuye hehe?
   4. Umaze igihe kingana gute utuye muri ako gace?
   5. Wigeze wiga?
   6. Ni uruhe rwego rw’amashuri wagarukiyeho??
   7. Wigeze ushaka, ubu warashatse cg waba uteganya gushakana n’umugore?

(Niba mutakibana mwamaranye igihe kinganiki? Niba ubu mubana, mumaranye igihe kinganiki?)

- 1. Ufite abana cyangwa waba uteganya kubagira?
  2. Ni iki ukora kikubeshejeho/kikwinjiriza?
  3. Hari umubano uhamye wigeze ugirana cg ufitanye n’umugabo?

1. Mbwira ku mibanire yawe n’aba bakurikira:
   1. Umufasha ( niba hari uhari)
   2. Abana ( niba hari abahari)
   3. Ababyeyi ( niba hari abahari)
   4. Abo muva inda imwe ( niba bahari)
   5. Abo mufite icyo mupfana (niba bahari)
   6. Inshuti (niba hari izihari)
   7. Abo mukorana ku kazi (niba bahari)
   8. Abo muhuje idini/imyemerere (niba bahari)
   9. Abandi (bavuge)
2. Wiyumva mu kihe cyiciro muri ibi bikurikira
3. Gay
4. Bisexual
5. Ikindi
6. Muri bariya bantu twavuze haruguru, hari abaziko uri umwe mu bagabo bagirana imibonano mpuzabitsina n’abandi bagabo?
7. Wigeze ushakana cyangwa ukundana n’umukobwa/umugore? Niba byarabaye iyo mibanire (Gushakana cyangwa gukundana biracyakomeza?)

**UBURYO BUBAHUZA**

1. Ugereranije hari abagabo bangahe bagirana imibonano mpuzabitsina n’abandi bagabo muri Kigali? Mu Rwanda?
2. Ni ubuhe buryo bukoreshwa kugira ngo ubashe kumenyana cyangwa no guhura n’abandi bagabo bagirana imibonano mpuzabitsina n’abandi bagabo?
3. Mbwira ku mashyirahamwe ahuza abagabo bagirana imibonano mpuzabitsina n’abandi bagabo ( Baza muri rusange, unabaze niba hari iryo abamo n’impamvu)
4. Tubwire ku mibanire/gukundana hagati y’abagabo bagirana imibonabo mpuzabitsina n’abandi bagabo

*(Baza muri rusange no ku giticye. Mushobora gukundana ku mugaragaro, mwisanzuye cg s mu ibanga? Sobanura)*

1. Hari ahantu cg ubundi buryo abagabo bagirana imibonano mpuzabitsina n’abandi bagabo bagira bubafasha guhura cg gusabana muri iki gihugu?
2. Nk’umwe mu bagabo bagirana imibonano mpuzabitsina n’abandi bagabo, mbwira icyo ubu buryo bukurikira bubafasha mu rwego rwo guhura no gusabana:
   1. Imbuga za murandasi zikoreshwa mu guhuza abagabo bagirana imibonano mpuzabitsina n’abandi bagabo
   2. Utubyiniro twihariye tw’abagabo
   3. Ibirori byihariye ku bagabo
   4. Ibindi birori
   5. Amahuriro y’urungano abakundana cg abashaka abakunzi bahuriramo
   6. Ubundi buryo
3. Ukurikije ubunararibonye bwawe mu ikoreshwa ry’uburyo bwavuzwe haruguru bw’abagabo bagirana imibonano mpuzabitsina n’abandi bagabo, ubusangiye n’abandi cyangwa urumva abandi bo bafite uburyo bwabo butandukanye n’ubwawe?
4. Ni iki giteye inkeke/impungenge mu bagabo bagirana imibonano mpuzabitsina n’abandi bagabo mu ikoresha ry’imbuga zihariye abagabo (MSM) bahuriraho
   1. Ubuzima bwite ( vie privée)
   2. Ibanga
   3. Umutekano
   4. Izindi nkurikizi

**IMYUMVIRE N’IMYITWARIRE Y’UMURYANGO NYARWANDA**

1. Muri rusange abagabo bagirana imibonano mpuzabitsina n’abandi bagabo bafatwa gute n’umuryango nyarwanda?
2. Kuri wowe by’umwihariko, ubona abantu baziko uri umwe mu bagabo bagirana imibonano mpuzabitsina n’abandi bagabo bagufata gute?
   1. Nk’umwe muri abo bagabo, bikugiraho izihe ngaruka zituruka k’uko umuryango nyarwanda ugufata?
   2. Bagenzi bawe bo bahura n’izihe ngaruka?
3. watubwira ubuzima bwawe ku bijyanye n’ihezwa cg akato wakorewe kuberako uri umwe mu bagabo bagirana imibonano mpuzabitsina n’abandi bagabo?
4. Ni gute umuryango Nyarwanda ugenda uhindura imyumvire n’uko ubanira abagabo bagirana imibonano mpuzabitsina n’abandi bagabo

**IMYITWARIRE N’UBUZIMA BUGENDANYE N’IMIBONANO MPUZABITSINA**

1. Muri iki gihe hari umuntu mugirana ibibonano mpuzabitsina? Tubwire niba uwo mubonana ari umugabo, umugore cyangwa ni bose?
2. Ni abantu bangahe ugereranije mwagiranye imibonano mpuzabitsina mu mezi atandatu ashize? Umwaka umwe ushize?
3. Ni izihe ngamba ufata zo kurinda ubuzima bwawe n’ubwo abandi igihe ukora imibonano mpuzabitsina? Tubwire kubijyanye n’uburyo bwo kwirinda indwara n’inda zitateguwe.
4. Waba warigeze ugira ikibazo cy’indwara zandurira mu myanya ndangagitsina (HIV n’izindi)? Tubwire igihe byabereye n’uko wabyikuyemo.
5. Iki cyaba ari ikibazo gikunze kugaragara kubagabo bagirana imibonano mpuzabitsina n’abandi bagabo? Ni ibihe bibazo bindi by’ubuzima mukunze guhura nabyo?
6. Hi hehe ujya kwivuriza iyo uhuye n’ikibazo cy’ubuzima? (Mbwira amazina yaho n’impamvu ariho uhitamo).
7. Abaganga b’aho wivuriza baba bazi ko uri umwe mubagirana imibonano mpuzabitsina n’abandi bagabo?
8. Ni izihe nzitizi abagabo bagirana imibonano mpuzabitsina n’abandi bagabo bakunze guhura nazo iyo bakeneye kwivuza cg ubundi bufasha butangirwa kwa muganga. ?
9. Ni iki waba uzi ku bijyanye na virus yitwa Human Papilloma (HPV)? (Baza, gerageza ubaze niba hari icyo ubazwa yaba yarumvise kuri iyo virus).
   1. Ni ikihe kibazo yaba itera mu bagabo bagirana imibonano mpuzabitsina n’abandi bagabo?
   2. Ni gute wabigenza igihe wamenya ko waba wanduye iyo virus?

Urakoze cyane kubw’umwanya wawe no kudusangiza ibitekerezo byawe uno munsi. Hari ikindi kintu wumva ushaka kutubwira tutigeze tuvugaho uno munsi?

**IBAZWA RIRARANGIYE. ZIMYA AKUMA GAFATA AMAJWI**
