# Supplementary material for: Social contexts as mediator of risk behaviors in Rwandan men who have sex with men (MSM): Implications for HIV and STI transmission
Source: PLoS One. 2019 Jan 18;14(1):e0211099. doi: 10.1371/journal.pone.0211099 (PMC6338414; doi:10.1371/journal.pone.0211099)
Supplement: S2 File — (DOCX) [file pone.0211099.s002.docx]

**Interview Guide for Men in Rwanda**

Thank you for your time today. My name is __________________________________________. I am part of a group of researchers here in Rwanda who are conducting an investigation into health issues affecting men in the MSM community here in Rwanda. Specifically, we are interested in understanding a variety of social and health behaviors including use of social media as well as knowledge of health issues, including human papillomavirus (HPV) and health seeking behavior. We have put together some questions to get insights your perception on these issues and how they affect men who have sex with men in Rwanda. We want to know how men in this community are empowered to deal with concerns around their health and wellbeing as well as factors that facilitate and/or hinder them in seeking medical care specifically around HPV. I will be asking you a few questions, some of which you may feel uncomfortable with. Please note that you are not under any obligation to answer any of the questions you are not comfortable with and feel free to stop me at any point you no longer wish to continue the interview.

Before we start, I would like to let you know that the information you will share with me today will be treated with utmost confidentiality and will be used only for research purposes. I would like to conduct this interview in a manner that protects your privacy as well as guarantee anonymity. Only authorized members of the research group will have access to the information you are giving me, which will only be used for research purposes. I will be using a digital device to record our conversation so I don't forget the important things you say to me. If you would prefer not have this interview digitally recorded, please let me know now. If you agree to everything I have said, I would need to obtain a verbal consent from you to proceed. Please state your consent on tape if you wish for this interview to continue. Thank you.

**BEGIN INTERVIEW. TURN ON DIGITAL RECORDER AND BRIEFLY INTRODUCE YOURSELF, PURPOSE OF INTERVIEW AND OBTAIN VERBAL CONSENT.**

**Socio-demographic**

1. Please introduce yourself. Feel free to use your real name, a nickname or pseudonym.
   1. When and where were you born?
   2. How long have you lived in this neighborhood?
   3. Have you been previously married, currently married or planning to marry?
   4. (If previously or currently married, probe for how long)
   5. Do you have or planning to have children?
   6. What do you do to earn a living/income?
2. Please tell me about your relationship with your:
   1. Spouse (if any)
   2. Children (if any)
   3. Parents
   4. Siblings
   5. Extended family
   6. Friends
   7. Colleagues at work
   8. Church or mosque affiliation
3. Are there people in the circles listed above who are aware of your status as a gay man?
4. Have you ever been married or in a relationship with a woman? Does this relationship still exist? (***Probe when ended if no longer existing***)

**MSM Social Networking**

1. How big is the gay community in this neighborhood? City? Country?
2. How and through what means do you identify and/or meet with other gay men in this community?
3. What is your relationship with other gay men in this community like?

(***Probe if open, cordial, restrictive, secretive and why?***)

1. What venues and/or opportunities exist for gay men to meet or socialize in this country?
2. Please describe your experiences using any and/or all of the following:
   1. Internet dating sites
   2. Men only (gentle men’s clubs
   3. Private men only parties
   4. Other social events
   5. Peer-peer network for meeting sexual partners
3. How similar or different would you say are the experiences of other gay men using each and/or all of the above means to connect and establish relationships?
4. When using social media are gay men concerned about:
   1. Privacy?
   2. Confidentiality?
   3. Safety?
   4. Any potential backlash?

**Social and Community Attitudes**

1. Are gay people generally accepted in this community?
2. What are your own experiences with regard to being accepted as a gay man by those who are aware of your preference for men as sexual partners?
   1. How does this affect your ability to identify yourself as a gay man?
   2. What about other gay men?
3. Tell me about your experiences of stigma and discrimination because you are gay
4. How has community perception and attitudes change toward gay people in this community
5. Sexual Behavior and Sexual Health
6. Are you currently in a sexual relationship? Please probe if relationship is with a man, woman or both?
7. How many sexual partners have you had in the last 6 months? One year?
8. What precautions do you take to maintain your health and wellbeing when having sexual intercourse? Probe for disease and/or pregnancy prevention methods
9. Have you ever had any sexual health problem (STIs, HIV, others)? Probe how long ago and actions taken to address the problem
10. Is this a common health problem among gay men? What other health problems are common among gay men?
11. Where do you usually go when you need health care?
    1. Specialist clinics for gay men
    2. Government health facilities
    3. Private health facilities
12. Are the medical staff in these facilities aware that you are a gay man?
13. What barriers exist for gay men seeking to improve their health and wellbeing in this community?
14. What do you know about Human Papillomavirus (HPV)? (Interviewer, describe if never heard of HPV)
    1. How does it affect gay men in this community?
    2. What would you do and where would you go if you suspect you have HPV?

Thank you for your time and for sharing your opinions with me today. Is there anything else you would like to talk about that we have not covered today?

**END INTERVIEW. TURN OFF DIGITAL RECORDER.**
